# Modified Aloe Polysaccharide Restores Chronic Stress-Induced Immunosuppression in Mice

**DOI:** 10.3390/ijms17101660

**Published:** 2016-09-30

**Authors:** Youngjoo Lee, Sun-A Im, Jiyeon Kim, Sungwon Lee, Junghak Kwon, Heetae Lee, Hyunseok Kong, Youngcheon Song, Eunju Shin, Seon-Gil Do, Chong-Kil Lee, Kyungjae Kim

**Affiliations:** 1 College of Pharmacy, Sahmyook University, Seoul 01795, Korea; jlyj777@gmail.com (Y.L.); shelly7285@naver.com (Ji.K.); leesw@live.co.kr (S.L.); bhonest@naver.com (Ju.K.); hite486@gmail.com (H.L.); hskong0813@gmail.com (H.K.); alexsongsu@syu.ac.kr (Y.S.); 2 College of Pharmacy, Chungbuk National University, Cheongju 28644, Korea; littlei@daum.net; 3 Wellness R&D Center, Univera, Inc., Seoul 04782, Korea; ejayshin@univera.com (E.S.); sgildo@univera.com (S.-G.D.)

**Keywords:** modified Aloe polysaccharide (MAP), electric foot shock (EFS), chronic stress, immune restoration, in vivo cytotoxic T lymphocyte (CTL)

## Abstract

Chronic stress generally experienced in our daily lives; is known to augment disease vulnerability by suppressing the host immune system. In the present study; the effect of modified Aloe polysaccharide (MAP) on chronic stress-induced immunosuppression was studied; this Aloe compound was characterized in our earlier study. Mice were orally administered with MAP for 24 days and exposed to electric foot shock (EFS; duration; 3 min; interval; 10 s; intensity; 2 mA) for 17 days. The stress-related immunosuppression and restorative effect of MAP were then analyzed by measuring various immunological parameters. MAP treatment alleviated lymphoid atrophy and body weight loss. The numbers of lymphocyte subsets were significantly normalized in MAP-treated mice. Oral administration of MAP also restored the proliferative activities of lymphocytes; ovalbumin (OVA)-specific T cell proliferation; antibody production; and the cell killing activity of cytotoxic T lymphocytes. In summary; oral administration of MAP ameliorated chronic EFS stress-induced immunosuppression.

## 1. Introduction

Stress is scientifically viewed as a natural and ubiquitous aspect of life; it is also referred to as the body’s adaptation to environmental threats [1,2]. For example, instantly exerted stress helps us adequately cope with emergencies by ensuring body functions are active. However, prolonged stress, which prevails in the present society, suppresses body functions and is indicated as a fundamental cause of numerous diseases [3].

Many studies have been conducted to identify this complex response since the 1970s [4]. In particular, the relationship between stress and the immune system has been discussed in depth. According to the stress-immune models theoretically established in the early 1980s, acute time-limited stress and chronic stress generate different immune reactions in our body [2]. Acute time-limited stress exerted under uncomfortable situations such as unexpected public speaking or memorization is associated with up-regulated immune functions including rapid movements of immune cells [5]. In contrast, chronic stress is considered broadly immunosuppressive. The stress hormones continuously produced under chronic stress conditions can disrupt immune cell responses particularly by altering patterns of cytokine secretion. This process results in the suppression of Type 1 helper T cells (Th1) with a subsequent increase in Type 2 helper T cells (Th2) response, rendering the defense system vulnerable to pathogenic infections and autoimmune diseases, respectively [6,7].

Many people today are suffering from this type of stress, experiencing stress induced-immunosuppression and vulnerability to diseases. Naturally, there is an increasing need for potential anti-stress agents, which help sustain a healthy immune system.

The Aloe plant species has long been studied as a prominent immune enhancer, and its immunomodulatory effects have been extensively reported, for example, improved lymphocyte proliferation, activation of complement, and anti-inflammatory effect [8,9]. There have been efforts to identify the active compound of Aloe plants. Additionally, the polysaccharide component of Aloe gel has been confirmed as a major immunomodulatory compound [10]. The size of the most active polysaccharide was further investigated. The processed Aloe consisting of polysaccharides ranging between 400 and 5 kDa, which is referred to as modified Aloe polysaccharide (MAP), exhibited potent immunomodulatory activities in previous studies [11,12]. For the present study, MAP was prepared by using the same method reported earlier with minor modifications [11,12]. The molecular weight of MAP was analyzed following the process; most of the polysaccharides ranged from 200 to 10 kDa.

The various immunomodulatory effects of MAP, such as anti-inflammation and antitumor, have been verified earlier in vitro and in vivo [11,12]; however, its effects on stress-induced immunosuppression have not been studied thus far. Therefore, we designed this study to determine the ameliorative effect of MAP on chronic electronic foot shock (EFS) stress-induced immunosuppression.

In the long history of stress studies with immune parameters, animal models have been exposed to various stressors such as electric foot shock (EFS), immobility, and low temperature [13,14] and EFS stimuli has been most commonly used to study stress-induced immunopathology [15,16]. Chronic stress in an animal study is generally characterized with repeated exposure to stressors on a daily basis for more than a week, whereas acute stress is a single application of the stressor [17,18]. For instance, the study conducted by Im and her co-workers exposed mice to EFS daily for 14 days and confirmed significant immune dysfunction, which was indicated by immune cell disturbance and suppressed proliferation [19]. Therefore, we designed experimental conditions based mainly on the previous studies, which successfully induced chronic stress [15,19]. Briefly, in the present study an experimental model of stress was developed by chronically exposing mice to EFS for 17 days with continuous oral administration of MAP. The protective effect of MAP against chronic EFS stress-induced immunosuppression in mice was then explored using various immunological parameters.

## 2. Results

### 2.1. Alleviation of Chronic Electric Foot Shock (EFS) Stress-Induced Body Weight Loss and Lymphoid Organ Atrophy by Modified Aloe Polysaccharide (MAP)

**Figure 1:**
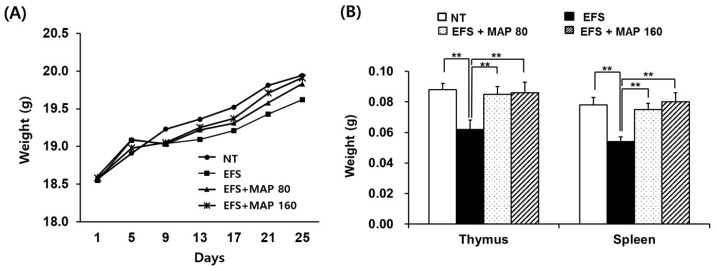
Alleviative effect of modified Aloe polysaccharide (MAP) against chronic electric foot shock (EFS) stress-induced atrophy of immune apparatus and weight loss. NT (No treatment), normal control; EFS only, EFS stress-induced mice; EFS + MAP 80, EFS stress-induced mice treated with a low dose (mg/kg) of MAP 80; EFS + MAP 160, EFS stress-induced mice treated with a high dose (mg/kg) of MAP 160. Average body weight (**A**) was measured every four days until sacrifice. The thymus and spleen were excised and weighed after sacrifice (**B**). Values are means ± SEM of three experiments, *n* = 20. ** *p* < 0.01, compared to control levels.

The body weight of mice exposed to EFS-mediated chronic stress decreased; these mice also developed lymphoid atrophy, which was evident in the weight indices of the thymus and spleen (Figure 1). However, the organs of the mice orally administered MAP were considerably protected and did not decrease in size. The alleviating effect of MAP on chronic EFS stress-induced lymphoid atrophy was marked in both the thymus and spleen and this effect appeared significant (Figure 1B) even when the average body weights were determined (Figure 1A).

### 2.2. Alleviative Effect of MAP on Chronic EFS Stress-Induced Disturbances in Lymphocyte Subsets

**Figure 2:**
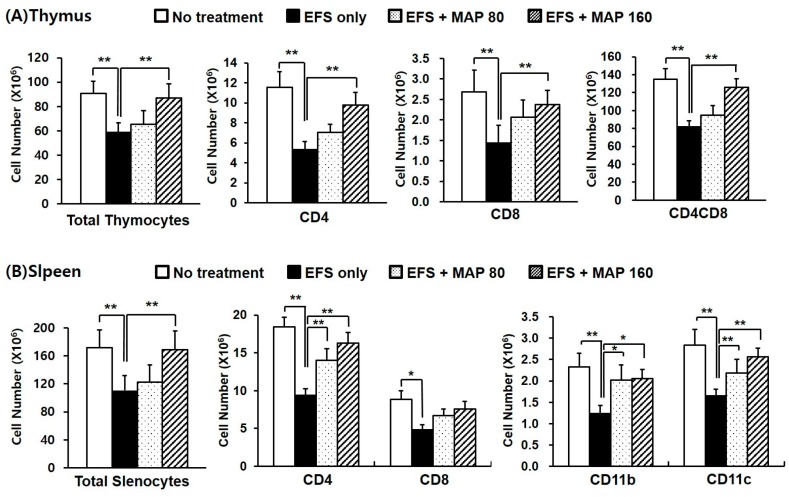
Protective effect of MAP against chronic EFS stress-induced disturbance in thymocyte and splenocyte cellularity. Mice are grouped as described in Figure 1. Lymphocyte subsets of the thymus (**A**) and spleen (**B**) were analyzed using flow cytometry, in which 10,000 cells were scored. Values are means ± SEM of three experiments, *n* = 7. * *p* < 0.05, ** *p* < 0.01 compared with control.

As shown in Figure 2, both the total cell numbers and the number of each lymphocyte subset were dramatically decreased in the thymus (Figure 2A) and spleen (Figure 2B) by chronic EFS stress, and this effect was considered to be related to the atrophy of the lymphoid organs. The thymocyte subsets—including CD4^+^, CD8^+^, and CD4^+^CD8^+^ lymphocytes—were all significantly protected by the high dose of MAP (160 mg/kg), whereas the splenocyte subsets—including CD4^+^, CD11b^+^, and CD11c^+^ lymphocytes—were significantly increased by administration of both doses of MAP (80 and 160 mg/kg).

### 2.3. Protective Effects of MAP against Chronic EFS Stress-Induced Suppression of Lymphocyte Proliferation

**Figure 3:**
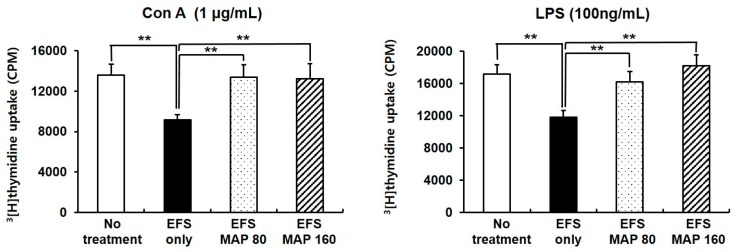
Protective effects of MAP on chronic EFS stress-induced suppression of lymphocyte proliferation. Mice are grouped as described in Figure 1. Isolated mice splenocytes were co-cultured with (**A**) Con A or (**B**) LPS. Proliferation of splenocytes was measured using ^3^(H)-thymidine incorporation assay. Values are means ± SEM of three experiments, *n* = 7. ** *p* < 0.01 compared with control.

Oral administration of MAP enhanced mitogen (Con A or LPS)-stimulated lymphocyte proliferation (Figure 3), which was impaired by chronic EFS stress. Furthermore, both doses of MAP (80 and 160 mg/kg) protected the proliferative function of lymphocytes.

### 2.4. Protective Effect of MAP against Chronic EFS Stress-Induced Disturbance in Lymphoid Organ Subsets of Ovalbumin (OVA)-Immunized Mice

**Figure 4:**
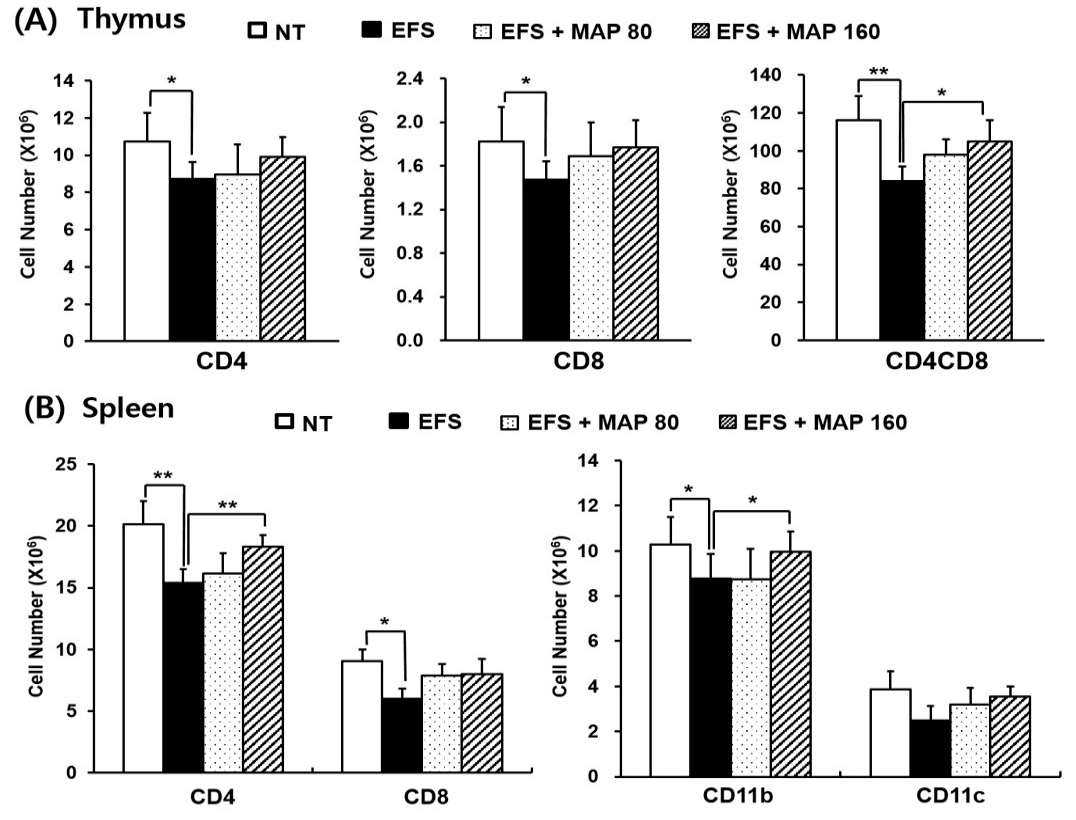
Protective effect of MAP against chronic EFS stress-induced disturbance of lymphocyte cellularity in OVA-immunized mice. Mice are grouped as described in Figure 1. Immunization with OVA was previously achieved in mice. Cellularity of lymphocytes in the (**A**) thymus and (**B**) spleen was analyzed using flow cytometry in which 10,000 cells were scored. Values are means ± SEM of three experiments, *n* = 5. * *p* < 0.05, ** *p* < 0.01 compared with control.

The number of each lymphocyte subset in both spleen and thymus was decreased by chronic EFS stress and significantly restored by MAP treatment, regardless of immunization (Figure 2 and Figure 4). When immunized, the CD4^+^CD8^+^ T lymphocytes were significantly protected by the high dose of MAP (160 mg/kg; Figure 4A), and the number of splenocyte subsets—including the CD4^+^ and CD11b^+^ lymphocytes—was significantly restored by the administration of high-dose MAP (Figure 4B). The absolute numbers of CD11b^+^ and CD11c^+^ cells, which are responsible for antigen processing, were considerably increased overall in all groups (Figure 4B) compared to those in non-immunized groups (Figure 2B), and the degree of decrease caused by EFS stress lessened in all subtypes after the immunization.

### 2.5. Immune Enhancing Effect of MAP on Immunoglobulin G (IgG) Production and Generation of OVA-Specific T Cells in Chronically Stressed Mice

**Figure 5:**
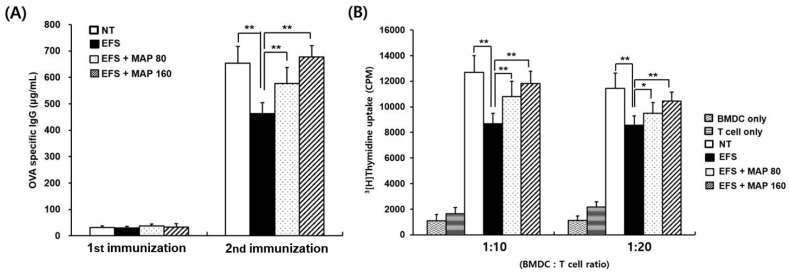
Immune enhancing effect of MAP on OVA-specific IgG production and OVA-specific T cell generation in chronically stressed mice. Mice are grouped as described in Figure 1. Immunization of OVA peptide was achieved in mice. Serum lgG levels of collected blood were monitored using ELISA analysis (**A**); Isolated T cells from OVA-immunized mice and OVA-pulsed bone marrow-derived cells (BMDCs) were co-cultured and DNA synthesis of T cells was measured using ^3^(H)-thymidine incorporation. T cells only, T cells from NT group, and BMDCs only, OVA-pulsed BMDCs were also separately cultured (**B**). Values are means ± SEM of three experiments, *n* = 5, * *p* < 0.05, ** *p* < 0.01 compared with control.

T cells isolated from the OVA-immunized spleen were co-cultured with p-OVA-pulsed bone marrow-derived cells (BMDCs), and the proliferative activity of T cells was examined on the last day of incubation. T cell proliferation was markedly weak in the chronic EFS stress-induced samples compared to that in those not subjected to stress. It is interesting to note that oral MAP administration protected cells against the effects of chronic EFS stress in a dose-dependent manner (Figure 5A).

Serum immunoglobulin G (IgG) levels of the primary and the secondary immunization were both presented in Figure 5B. The OVA-specific IgG production induced by the initial immunization was marginal in all experimental groups because IgG production preceded IgM production. However, following the secondary booster immunization, substantial amounts of specific IgG were detected in all groups. The results showed that chronic EFS stress largely reduced the specific IgG production, whereas oral administration of MAP enhanced antibody productivity in a dose-dependent manner (Figure 5B).

### 2.6. Immune Enhancing Effect of MAP on Generation of Cytotoxic T Lymphocyte (CTL) in Chronically Stressed Mice

**Figure 6:**
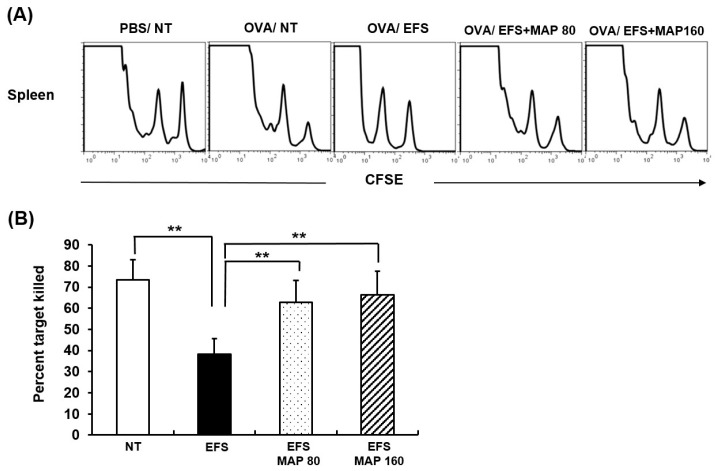
Immune enhancing effect of MAP on activity of cytotoxic T lymphocytes (CTLs) in chronically stressed mice. Mice are grouped as described in Figure 1 and were OVA-immunized. (**A**) Histogram peaks show number of target cells in untreated PBS-immunized group, determined to indicate level of two target cell populations; (**B**) Specific killing (%) of OVA (257–264) peptide-pulsed target cells in the spleen. Ratio of carboxyfluorescein succinimidyl ester (CFSE)_high_ and CFSE_low_ was calculated as a numerical value to present specific killing. Values are means ± SEM of three experiments, *n* = 5, ** *p* < 0.01 compared with control.

OVA-specific CTL killing function in vivo was presented in two forms; visually in histograms (A) and using absolute numerical values which were calculated as killing ratios (B). The two peaks in the histograms (A) of Figure 6 represent OVA-coated cells and uncoated one respectively, thus we can assume the OVA-specific CTL killing activity by comparing these two peaks. As indicated by the first histogram, specific cell death was achieved by CTL only in the OVA-immunized and not in PBS-immunized mice. It is a significant finding that the generation of the potent CTL was notably suppressed by chronic EFS stress, whereas oral MAP administration prevented this deteriorative effect. These results are presented as percentages in Panel (B) of Figure 6.

## 3. Discussion

The majority of people today have been experiencing health deterioration because of exposure to chronic stress. Chronic stress is known to contribute significantly to the development of various diseases such as ulcer, cancer metastasis, and cardiovascular disease [20,21,22], which has been explicated with its detrimental effects on host immune systems [23,24]. Keeping our immune system robust can be a significant strategy for stress management, and we can make ourselves resistant to chronic stress by greatly nourishing our immunity with the use of immune stimulants on a regular basis. Immune stimulants will reduce the risk of diseases by buffering the chronic stress induced-immunosuppression.

The Aloe compound, especially MAP, is one of the promising candidates from this perspective. According to previous studies [11,12] in which the bioactive effect of MAP on mice in vivo had already been investigated, the treatment conditions as appropriate concentrations were designated for the present study. To exhibit maximum bio-activities of MAP in the host immune system, the oral administration of MAP was initiated seven days earlier than stress exposure [15,16,19].

As described above, successful induction of chronic stress in mice was confirmed with various immunological indexes including immune cell distribution and lymphocyte functions. The effect of MAP was then evaluated based on whether it improved those parameters or not.

The EFS-induced chronic stress caused significant lymphoid atrophy, which was restored by the oral administration of MAP (Figure 1B). The atrophy in the immune apparatus has been commonly observed in numerous experimental stress models, reflecting the host’s immune conditions [25].

We obtained consistent results at the cellular level, following characterization of the isolated lymphocytes from the lymphoid organs. The number of major lymphocyte subsets were consistently decreased by EFS stress and restored by MAP treatment (Figure 2). This pattern was also confirmed when the hosts were exposed to an external antigen (Figure 4). It is necessary that each immune cell type is balanced in the appropriate number for proper function of the immune system. CD4^+^ T cell is crucial to the overall immune regulation because it determines the appropriate immune responses to each antigen (whether to exhibit a cellular or humoral reaction) [26] and CD8^+^ T cell operates the essential defense mechanism for virus or tumors, being responsible for the cellular immunity. CD11b^+^ and CD11c^+^ cells, professional antigen presenting cells such as dendritic cells and macrophages, represent the host innate immunity, which confronts and handles external antigens directly [26]. In this context, the restorative effects of MAP on the numbers of lymphocyte subsets are quite significant, especially CD4^+^CD8^+^ immature T cells in thymus, given that immature lymphocytes are the majority in the thymus and they are known to be much more fragile to stress [27].

On the other hand, not only the normal lymphocytes but also the immunized lymphocytes were analyzed for their functional capability. First, the total splenocytes isolated from normal mice were cultured for the proliferation assay in which the lymphocyte was induced by LPS or Con A, a mitogen for B or T cells, respectively (Figure 3). The susceptibility of lymphocytes to these mitogens was presented as the degree of proliferation, which can be interpreted as their functional properties. The impaired proliferative activity of the lymphocyte induced by EFS-mediated chronic stress was significantly improved with MAP treatment (Figure 3).

Second, the proliferation of OVA-specific T cells was measured, which was also greatly suppressed by chronic EFS stress and ameliorated by MAP treatment (Figure 5A). The T cells were exposed to the OVA antigen in vivo and then co-cultured with OVA-presenting BMDCs in vitro. Therefore, this proliferation assay can determine the ability of helper T cells to recognize antigens and mediate specific immunity.

In addition to specific T cell proliferation assay, the specific antibody production level was examined. The value obtained represents the immune function mediated by the humoral response, which is one of the efficient defense strategies in combination with cellular responses [26]. The decrease in the IgG level of EFS-induced chronically stressed mice was successfully prevented by oral administration of MAP (Figure 5B).

Lastly, the specific cell-killing potential of the cytotoxic T cells was also assessed using in vivo CTL assay, by which host cellular response against antigens can be assumed. MAP treatment practically enabled the cytotoxic T cells to exert their killing functions, which were suppressed by chronic EFS stress (Figure 6).

## 4. Materials and Methods

### 4.1. Preparation of MAP

MAP was prepared as described previously with minor modifications [12]. In brief, the native *Aloe vera* gel was subjected to cellulose treatment to maximize the extraction of polysaccharide, and the protein components were removed by passaging through a DEAE-Sephacel column. To obtain the polysaccharide of optimal size, the protein-free Aloe materials were further separated by Sephacryl column chromatography and filtration. Lastly, MAP was refined by passing through a dialysis membrane; the resulting MAP was free of low molecular fractions (<3.5 kDa). The lyophilized MAP was suspended in saline to obtain stock solutions of 16 and 32 mg/mL.

### 4.2. Animals and Experimental Treatments

Male six-week-old C57BL/6 mice were purchased from Orient Bio Co., (Seoul, Korea) and allowed to acclimatize for one week. The mice were housed in a laboratory animal facility at 20–24 °C, humidity of 30%–70%, a 12-h light-dark cycle, and free access to commercial rodent chow and sterile water. All experimental procedures were performed in strict compliance with the Guidelines for the Care and Use of Laboratory Animals issued by Sahmyook University (IACUC number: SYUIACUC 2014040, 1 November 2014).

As described above, the stress model associated with immunosuppression was designed following the previous protocol [19]. In this study, the mice were exposed to EFS (duration, 3 min; interval, 10 s; intensity, 2 mA) daily for 17 days and orally administrated with MAP in two different doses (80 and 160 mg/kg) for 24 days, which was initiated 7 days prior to EFS induction. On day 25, the mice were euthanized using ether.

### 4.3. Lymphocyte Subset Analysis

Single cells were isolated from spleen and thymus and stained with monoclonal antibodies for immune cell phenotyping as previously described [28]. Briefly, after isolating the single cells, their non-specific binding was inhibited by blocking Fc receptors, and then the lymphocytes were stained with monoclonal antibodies; anti-CD4 (clone GK1.5), anti-CD8 (clone 53–6.7), anti-CD11b (clone M1.70) and anti-CD11c (N418) (BD biosciences, Franklin Lakes, NJ, USA). Lastly, the stained cells were fixed with 1% paraformaldehyde in phosphate-buffered solution (PBS). Approximately 10,000 cells from each sample were analyzed using a fluorescence-activated cell sorting (FACS) Caliver system (Becton, Dickinson and Company, Franklin Lakes, NJ, USA).

### 4.4. Lymphocyte Proliferation Assay

Splenocytes were cultured under the general cell culture condition [19] and incubated in the presence of a mitogen such as Con A (1 µg/mL) or lipopolysaccharide (LPS; 100 ng/mL) and their proliferative activity was assessed using a ^3^(H)-thymidine incorporation assay. A solution containing 1 µCi of ^3^(H)-thymidine was added to each well and incubated for an additional 16 h until the total incubation period was 72 h. Then, the cultured cells were harvested and transferred onto a glass filter, which was placed in a sample bag containing scintillation cocktail. The level of ^3^(H)-thymidine incorporated was measured using a microbeta counter (Wallac, Waltham, MA, USA).

### 4.5. Measurement of Immunoglobulin G (IgG) Concentration in Peripheral Blood

We induced mice in each group to produce IgG; production was initiated by the secondary immunization of a certain antigen. To achieve antigen-specific immunization, the mice were administered subcutaneously with ovalbumin (OVA) peptide dissolved in complete Freud’s adjuvant (CFA, Sigma-Aldrich Corp., St. Louis, MO, USA) after seven days of MAP treatment. A secondary injection of OVA peptide in incomplete Freud’s adjuvant (IFA, Sigma-Aldrich Corp., St. Louis, MO, USA) was performed eight days after the primary injection.

Following each immunization, peripheral blood samples were collected by retro-orbital bleeding of the live mice. Serum from the collected blood samples was used to monitor the IgG levels using an enzyme-linked immunosorbent assay (ELISA) analysis. An Immuno-plate (NUNC, Roskilde, Denmark) was coated overnight with OVA solution (in PBS, Sigma-Aldrich Corp.), followed by blocking with 10% fetal bovine saline (FBS) in PBS. The serum samples and standard were placed onto the plate, incubated, and then horseradish peroxidase (HRP)-conjugated goat anti-mouse IgG antibodies (Sigma-Aldrich, Corp.) were added for specific binding, followed by 3,3′,5,5′-tetramethybenzidine (TMB) substrate solution and stop solution to detect the lgG concentration.

### 4.6. Proliferation Assay of OVA-Specific T Lymphocytes

Spleen cells were harvested from OVA-immunized mice as described above. The splenocyte suspension in Dulbecco’s modified Eagle’s medium (DMEM, Hyclone) was incubated in a nylon wool column to isolate only T cells. In addition, bone marrow-derived cells (BMDCs) were prepared from normal mice for co-culturing. The BMDCs were induced to differentiate into macrophages or dendritic cells by incubation with granulocyte macrophage colony stimulating factor (GM-CSF; 40 ng/mL) for six days. After the culture period, BMDCs were harvested and further cultured in the presence of p-OVA (50 µg/mL) at 37 °C. The isolated T cells and OVA-pulsed BMDCs were then co-cultured in a 10:1 or 20:1 ratio for three days. On the last culture day, the proliferation of OVA-specific T cells was measured using a ^3^(H)-thymidine incorporation assay as previously described.

### 4.7. In Vivo Cytotoxic T Lymphocyte (CTL) Assay

According to the method performed by Im et al. [29], mice were OVA-immunized by intravenous injections of soluble OVA (100 µg/mouse, Sigma-Aldrich Corp.) and cytotoxic T lymphocyte (CTL) functions were examined seven days later using flow cytometry. In brief, splenocytes from naive syngeneic mice were prepared as target cells, which were pulsed with OVA (257–264) peptide, and then labeled with a high concentration of carboxyfluorescein succinimidyl ester (CFSE, 5 µM, CFSE_high_). In addition, a similar quantity of unpulsed syngeneic cells was prepared and labeled with a low concentration of CFSE (1 µM, CFSE_low_) as the control. A 1:1 mixture of each target cell population (1 × 10^7^ cells/mouse) was injected via the tail vein of OVA-immunized mice. The recipient mice were euthanized following the injection, and then their splenocytes were analyzed using flow cytometry.

### 4.8. Statistical Analysis

The results are presented as mean ± standard error of the mean (SEM) and the significant differences between the control and treated groups were statistically analyzed using a one-way analysis of variance (ANOVA) followed by a Tukey’s test.

## 5. Conclusions

The results presented above consistently suggest that oral administration of MAP markedly prevented the immune system from being weakened under chronic EFS stress conditions. Furthermore, the immune-enhancing effect of MAP was demonstrated on every component of the immune system not only on the non-specific and specific immunity, but also on cellular and humoral responses of the specific immunity.

## References

[1] Dhabhar F.S. (2014). Effects of stress on immune function: The good, the bad, and the beautiful. Immunol. Res..

[2] Segerstrom S.C., Miller G.E. (2004). Psychological stress and the human immune system: A meta-analytic study of 30 years of inquiry. Psychol. Bull..

[3] Dhabhar F.S. (2002). A hassle a day may keep the doctor away: Stress and the augmentation of immune function. Integr. Comp. Biol..

[4] Selye H. (1977). A code for coping with stress. Aorn. J..

[5] Dhabhar F.S., Mcewen B.S. (1997). Acute stress enhances while chronic stress suppresses cell-mediated immunity in vivo: A potential role for leukocyte trafficking. Brain Behav. Immun..

[6] Marshall G.D., Agarwal S.K., Lloyd C., Cohen L., Henninger E.M., Morris G.J. (1998). Cytokine dysregulation associated with exam stress in healthy medical students. Brain Behav. Immun..

[7] Chiappelli F., Manfrini E., Franceschi C., Cossarizza A., Black K.L. (1994). Steroid regulation of cytokines: Relevance for TH1-to-TH2 shift?. Ann. N. Y. Acad. Sci..

[8] Balan B.J., Niemcewicz M., Kocik J., Jung L., Skopinska-Rozewska E., Skopinski P. (2014). Oral administration of *Aloe vera* gel, anti-microbial and anti-inflammatory herbal remedy, stimulates cell-mediated immunity and antibody production in a mouse model. Cent. Eur. J. Immunol..

[9] Hart L.A., van den Berg A.J., Kuis L., van Dijk H., Labadie R.P. (1989). An anti-complementary polysaccharide with immunological adjuvant activity from the leaf parenchyma gel of *Aloe vera*. Planta Med..

[10] Reynolds T., Dweck A.C. (1999). *Aloe vera* leaf gel: A review update. J. Ethnopharmacol..

[11] Qiu Z., Jones K., Wylie M., Jia Q., Orndorff S. (2000). Modified Aloe barbadensis polysaccharide with immunoregulatory activity. Planta Med..

[12] Im S.A., Oh S.T., Song S., Kim M.R., Kim D.S., Woo S.S., Jo T.H., Park Y.I., Lee C.K. (2005). Identification of optimal molecular size of modified Aloe polysaccharides with maximum immunomodulatory activity. Int. Immunopharmacol..

[13] Lysle D.T., Lyte M., Fowler H., Rabin B.S. (1987). Shock-induced modulation of lymphocyte reactivity: Suppression, habituation, and recovery. Life Sci..

[14] Batuman O.A., Sajewski D., Ottenweller J.E., Pitman D.L., Natelson B.H. (1990). Effects of repeated stress on T cell numbers and function in rats. Brain Behav. Immun..

[15] Zhao T.T., Shin K.S., Choi H.S., Lee M.K. (2015). Ameliorating effects of gypenosides on chronic stress-induced anxiety disorders in mice. BMC Complement. Altern. Med..

[16] Choi H.S., Park M.S., Kim S.H., Hwang B.Y., Lee C.K., Lee M.K. (2010). Neuroprotective effects of herbal ethanol extracts from *Gynostemma pentaphyllum* in the 6-hydroxydopamine-lesioned rat model of Parkinson’s disease. Molecules.

[17] Sutanto W., de Kloet E.R. (1994). The use of various animal models in the study of stress and stress-related phenomena. Lab. Anim..

[18] Bhatia N., Maiti P.P., Choudhary A., Tuli A., Masih D., Masih M., Khan U., Ara T., Jaggi A.S. (2011). Animal models in the study of stress: A review. NSHM J. Pharm. Healthc. Manag..

[19] Im S.A., Choi H.S., Choi S.O., Kim K.H., Lee S., Hwang B.Y., Lee M.K., Lee C.K. (2012). Restoration of electric footshock-induced immunosuppression in mice by *Gynostemma pentaphyllum* components. Molecules.

[20] McEwen B.S. (2002). Protective and damaging effects of stress mediators: The good and bad sides of the response to stress. Metabolism.

[21] Ben-Eliyahu S., Yirmiya R., Liebeskind J.C., Taylor A.N., Gale R.P. (1991). Stress increases metastatic spread of a mammary tumor in rats: Evidence for mediation by the immune system. Brain Behav. Immun..

[22] Black P.H., Garbutt L.D. (2002). Stress, inflammation and cardiovascular disease. J. Psychosom. Res..

[23] Dhabhar F.S. (2009). Enhancing versus suppressive effects of stress on immune function: implications for immunoprotection and immunopathology. Neuroimmunomodulation.

[24] Dhabhar F.S., Malarkey W.B., Neri E., McEwen B.S. (2012). Stress-induced redistribution of immune cells—From barracks to boulevards to battlefields: A tale of three hormones—Curt Richter Award winner. Psychoneuroendocrinology.

[25] Wang K.X., Denhardt D.T. (2008). Osteopontin: Role in immune regulation and stress responses. Cytokine Growth Factor Rev..

[26] Parham P. (2005). The Immune System.

[27] Purton J.F., Monk J.A., Liddicoat D.R., Kyparissoudis K., Sakkal S., Richardson S.J., Godfrey D.I., Cole T.J. (2004). Expression of the glucocorticoid receptor from the 1A promoter correlates with T lymphocyte sensitivity to glucocorticoid-induced cell death. J. Immunol..

[28] Lee Y.H., Lee Y.R., Im S.A., Park S.I., Kim K.H., Gerelchuluun T., Song S., Kim K., Lee C.K. (2007). Calcineurin inhibitors block MHC-restricted antigen presentation in vivo. J. Immunol..

[29] Im S.A., Lee Y.R., Lee Y.H., Lee M.K., Park Y.I., Lee S., Kim K., Lee C.K. (2010). In vivo evidence of the immunomodulatory activity of orally administered *Aloe vera* gel. Arch. Pharm. Res..

